# Supplementary material for: Extracting interpretable signatures of whole-brain dynamics through systematic comparison
Source: bioRxiv. 2024 Jun 10:2024.01.10.573372. Preprint. [Version 2] doi: 10.1101/2024.01.10.573372 (PMC11195072; doi:10.1101/2024.01.10.573372)
Supplement: 1 [file NIHPP2024.01.10.573372V2-supplement-1.pdf]

bioRxiv preprint doi: https://doi.org/10.1101/2024.01.10.573372; this version posted June 10, 2024. The copyright holder for this preprint (which was not certified by peer review) is the author/funder, who has granted bioRxiv a license to display the preprint in perpetuity. It is made available under aCC-BY 4.0 International license.

Supplementary figures for "Extracting interpretable signatures of whole-brain dynamics through systematic comparison"

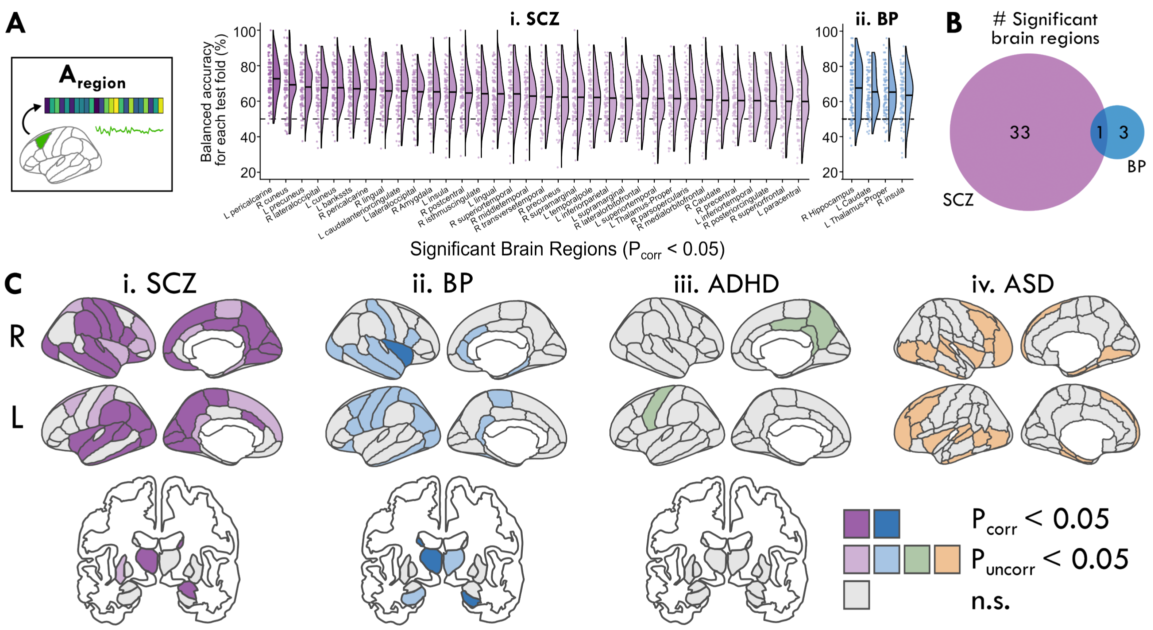

Figure S1. While several brain regions exhibited altered dynamics in each case–control comparison, only a subset were significant in SCZ and BP after multiple comparisons. A. The distribution of balanced accuracy values across test folds is shown as a raincloud plot for each significant brain region ($P_{adj} < 0.05$, corrected across 82 regions) in SCZ (**i**) and BP (**ii**). The horizontal line within each half-violin indicates the mean balanced accuracy for the corresponding brain region. B. The Venn diagram illustrates the number of significant brain regions for each of SCZ (purple) and BP (blue), indicating that one brain region (left thalamus) is shared between the two disorders. **C.** For each of the four disorders, regions are shaded dark to indicate $P_{adj} < 0.05$ (corrected across 82 regions for SCZ, BP, and ADHD; across 48 regions for ASD). Additionally, regions are shaded light to indicate that the nominal uncorrected $P < 0.05$. Gray shading indicates that the uncorrected $P > 0.05$ for the balanced accuracy in the given region.

bioRxiv preprint doi: https://doi.org/10.1101/2024.01.10.573372; this version posted June 10, 2024. The copyright holder for this preprint (which was not certified by peer review) is the author/funder, who has granted bioRxiv a license to display the preprint in perpetuity. It is made available under aCC-BY 4.0 International license.

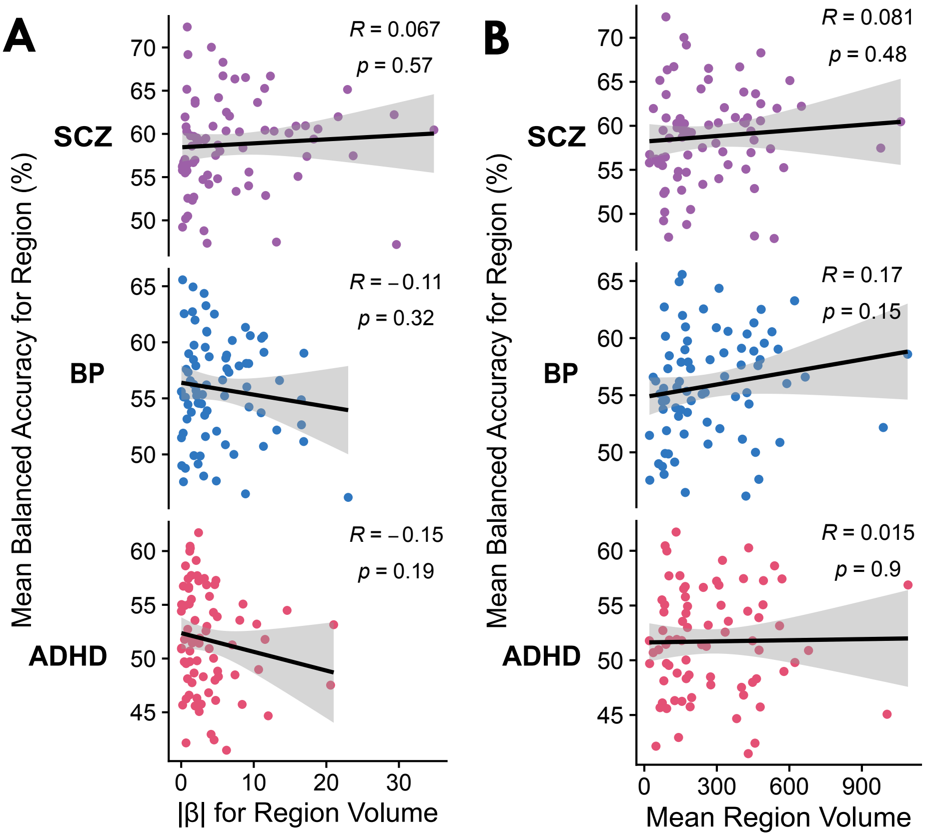

Figure S2. Region-wise classification balanced accuracy is not associated with volumetric differences across clinical groups. A. For each brain region in the UCLA CNP dataset, the mean balanced accuracy is plotted relative to the absolute β coefficient estimated from ordinary least squares regression of region volume on diagnosis per clinical group. Pearson correlation estimates (R) and corresponding P-values are annotated in the top right corners. **B.** As in A, for each brain region, the mean balanced accuracy is plotted relative to the average region volume (measured in number of voxels) across all participants in the UCLA CNP cohort. Pearson correlation estimates, R, and corresponding P-values are shown in the top right corners.

bioRxiv preprint doi: https://doi.org/10.1101/2024.01.10.573372; this version posted June 10, 2024. The copyright holder for this preprint (which was not certified by peer review) is the author/funder, who has granted bioRxiv a license to display the preprint in perpetuity. It is made available under aCC-BY 4.0 International license.

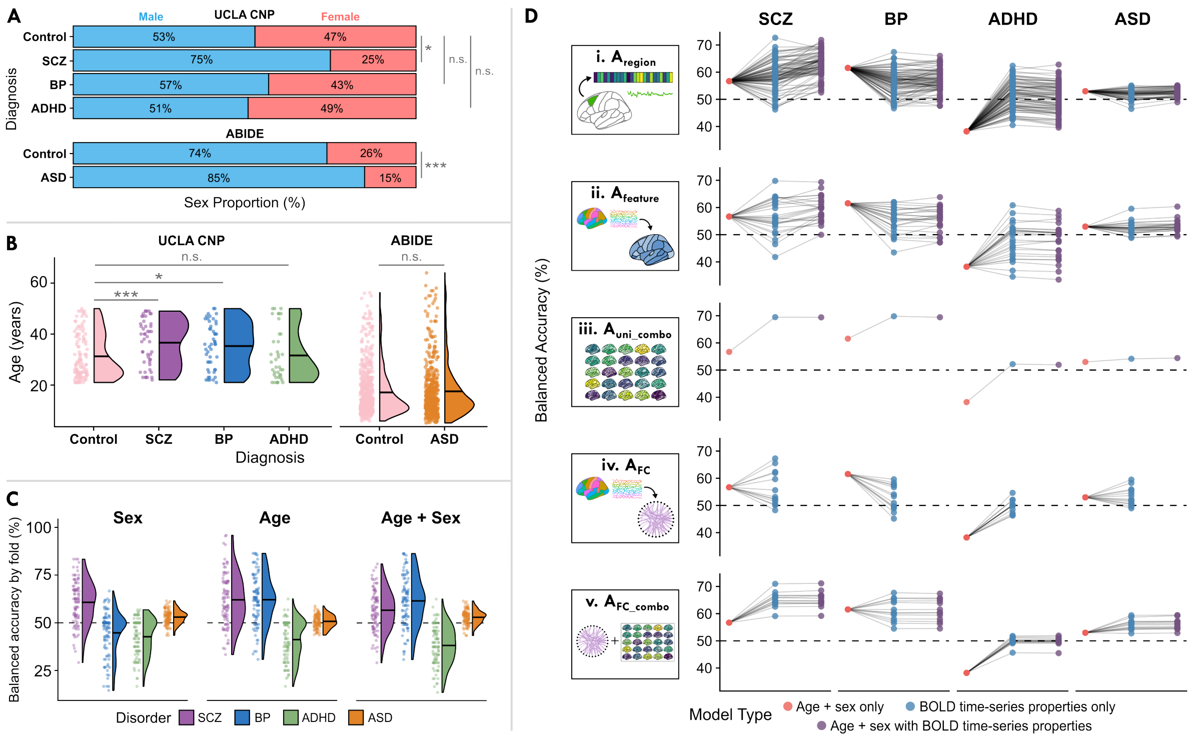

Figure S3. Feature-based representations improve diagnostic classification beyond age and sex. A. The percentage of males (blue) and females (red) per diagnostic group is shown for the UCLA CNP cohort (upper) and the ABIDE cohort (lower). Sex proportions were compared for each disorder relative to the corresponding control group using a chi-square test, with significance level indicated as $^{***}P < 0.001$, $^{**}P < 0.01$, $^{*}P < 0.05$, n.s. $P > 0.05$. **B.** The distribution of participant ages is shown as a raincloud plot per diagnostic group for the UCLA CNP cohort (left) and the ABIDE cohort (right). The horizontal line within each half-violin indicates the mean age for the corresponding distribution. Age distributions were compared for each disorder relative to the corresponding control group using a Wilcoxon rank-sum test, with significance level indicated as $^{***}P < 0.001$, $^{**}P < 0.01$, $^{*}P < 0.05$, n.s. $P > 0.05$. **C.** Case–control classification balanced accuracy is shown for all 100 test folds per each disorder based on participant sex, age, or the combination of age and sex data. The horizontal line within each half-violin indicates the mean balanced accuracy for the corresponding distribution. **D.** For each representation type (rows) and neuropsychiatric disorder (columns), the classification balanced accuracy using just age + sex data (red) is compared with each BOLD time-series feature model (blue) or the combination of BOLD time series features plus age and sex (purple). Each dot corresponds to one model (e.g., the left pericalcarine cortex in [i] A_{region}) and lines connect model types to guide visual comparison. The horizontal dashed line in each plot is included to show the chance baseline performance of 50% balanced accuracy.

bioRxiv preprint doi: https://doi.org/10.1101/2024.01.10.573372; this version posted June 10, 2024. The copyright holder for this preprint (which was not certified by peer review) is the author/funder, who has granted bioRxiv a license to display the preprint in perpetuity. It is made available under aCC-BY 4.0 International license.

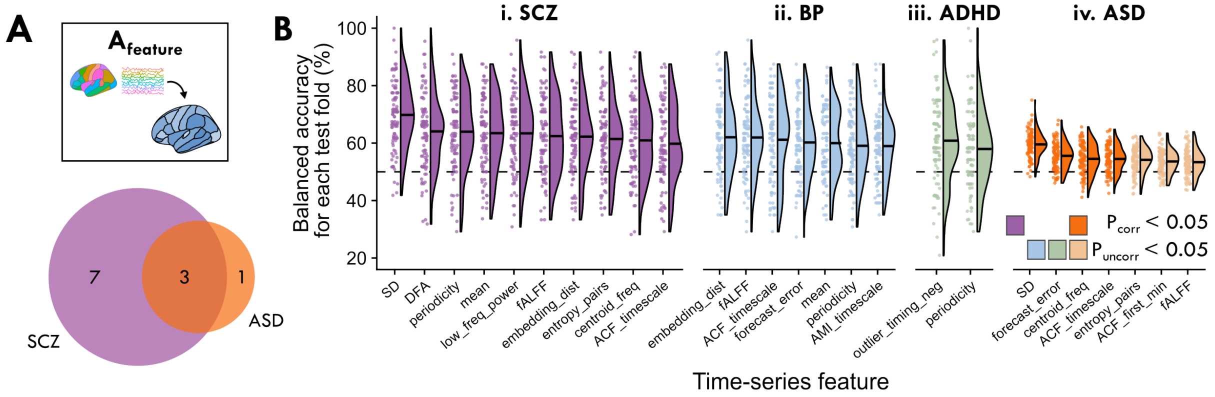

Figure S4. While several brain regions exhibited altered dynamics in each case–control comparison, only a subset were significant in SCZ and BP after multiple comparisons. A. The Venn diagram illustrates the number of significant intra-regional time-series features for each of SCZ (purple) and ASD (orange), indicating that three features are shared between the two disorders. B. The distribution of balanced accuracy values across test folds is shown as a raincloud plot for each intra-regional time-series feature that yielded a balanced accuracy with either $P_{\mathrm{corr}} < 0.05$ (darker) or $P_{\mathrm{uncorr}} < 0.05$ (lighter). The horizontal line within each half-violin indicates the mean balanced accuracy for the intra-regional time-series feature.

bioRxiv preprint doi: https://doi.org/10.1101/2024.01.10.573372; this version posted June 10, 2024. The copyright holder for this preprint (which was not certified by peer review) is the author/funder, who has granted bioRxiv a license to display the preprint in perpetuity. It is made available under aCC-BY 4.0 International license.

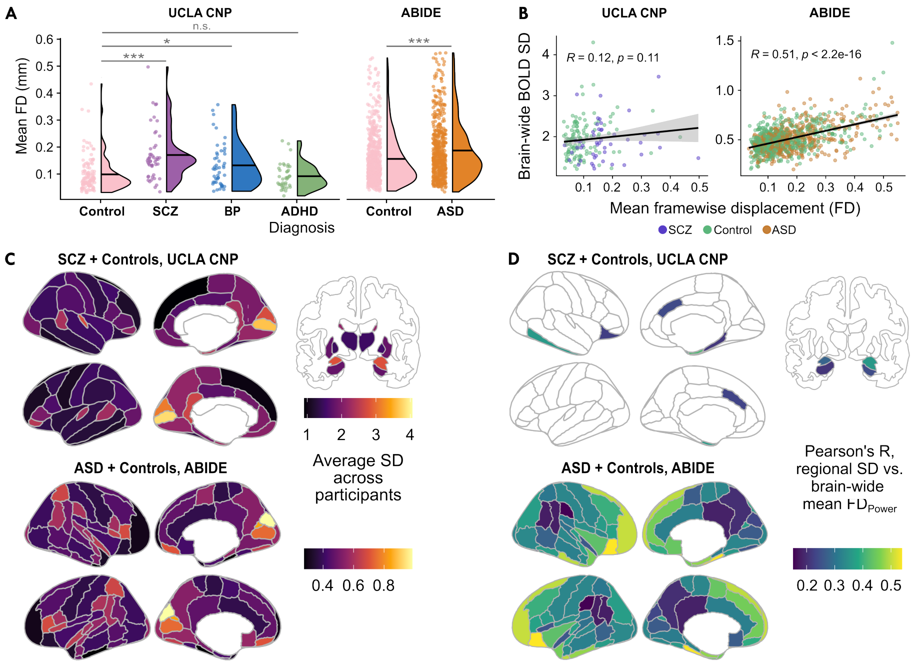

Figure S5. Head motion is generally higher in cases than in controls, but is only associated with brain-wide BOLD SD in the ABIDE cohort. A. The mean framewise displacement (FD) computed with the method from Power et al. [170] is shown with raincloud plots for all participants in the UCLA CNP (left) and ABIDE cohorts (right). The horizontal line within each half-violin indicates the mean FD for the corresponding group. Mean FD distributions were compared for each disorder relative to the corresponding control group using a Wilcoxon rank-sum test, with significance level indicated as $^{***}P < 0.001$, $^{**}P < 0.01$, $^{*}P < 0.05$, n.s. $P > 0.05$. **B.** The brain-wide average BOLD SD is plotted against the mean FD across SCZ and control participants in the UCLA CNP cohort (upper) as well as ASD and control participants in the ABIDE cohort (lower), with the Pearson correlation estimates (R) and corresponding p-values shown in each plot. **B.** For each brain region, the average BOLD SD is shown across all SCZ an control participants in the UCLA CNP cohort (upper) as well as ASD an control participants in the ABIDE cohort (lower). Note that different color scales are used for the two cohorts, respectively. **D.** For each brain region, we computed the Pearson correlation between the region-wise BOLD SD and whole-brain mean FD values in the UCLA CNP cohort (upper) as well as ASD and control participants in the ABIDE cohort (lower). Pearson correlation estimates (R) are shown in brain maps, in which only brain regions for which Benjamini–Hochberg corrected $P < 0.05$ are shaded (corrected across 82 regions for UCLA CNP and 48 regions for ABIDE). Note that the same color scale is used for both cohorts.

bioRxiv preprint doi: https://doi.org/10.1101/2024.01.10.573372; this version posted June 10, 2024. The copyright holder for this preprint (which was not certified by peer review) is the author/funder, who has granted bioRxiv a license to display the preprint in perpetuity. It is made available under aCC-BY 4.0 International license.

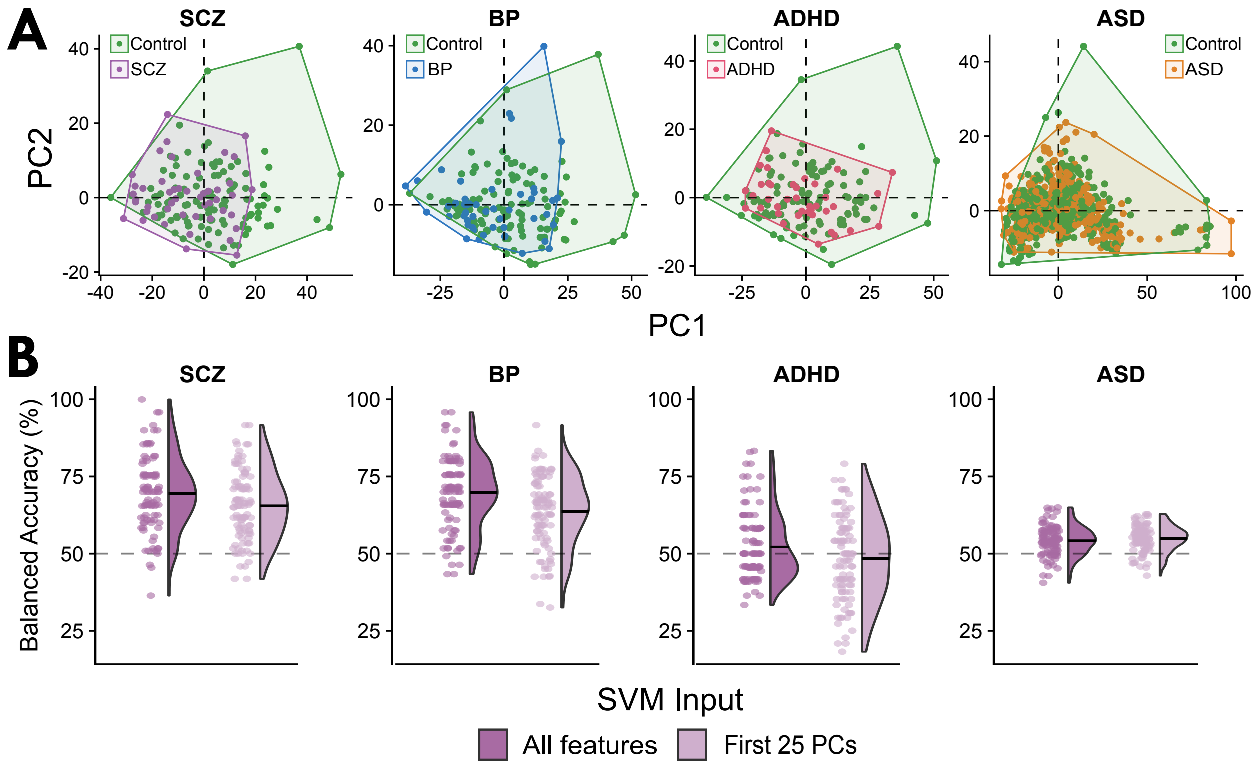

Figure S6. Linear dimensionality reduction and regularization approaches did not improve out-of-sample classification for the univariate region × feature classifiers, $\mathbf{A_{uni\_combo}}$. A. For each disorder, individual scores for the first two PCs are plotted, with points colored according to diagnosis. Shaded areas reflect convex hulls encapsulating all points for each diagnostic group. Note that each PCA was computed separately for each case–control comparison, so PC1 and PC2 scores are not directly comparable across clinical groups. **B.** For each case–control comparison, we compare the out-of-sample balanced accuracy across the 100 repeats × folds using all region × feature variables (left, dark purple) versus using only scores for the first 25 PCs (right, light purple). Points are randomly jittered along the horizontal axis in each raincloud plot to aid visualization. The horizontal line within each half-violin indicates the mean balanced accuracy for the corresponding distribution. **C.** For each case–control comparison, we compare the out-of-sample balanced accuracy across the 100 repeats×folds using default regularization (left, dark green) versus L1 ('LASSO' [186]) regularization (right, light green).

bioRxiv preprint doi: https://doi.org/10.1101/2024.01.10.573372; this version posted June 10, 2024. The copyright holder for this preprint (which was not certified by peer review) is the author/funder, who has granted bioRxiv a license to display the preprint in perpetuity. It is made available under aCC-BY 4.0 International license.

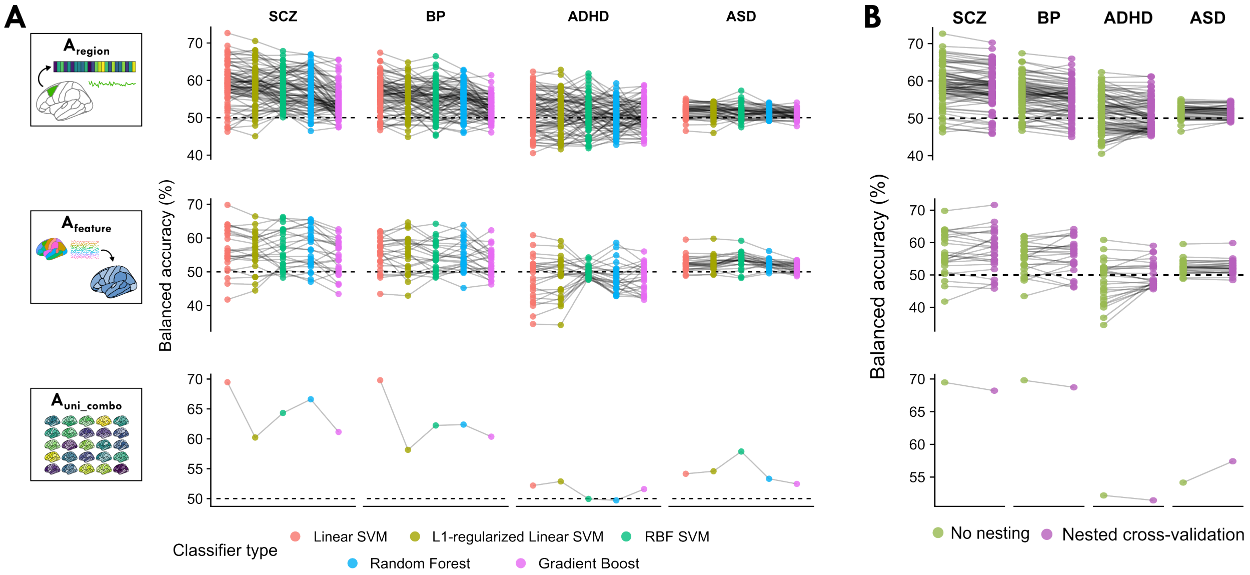

Figure S7. Comprehensive comparison of classifier types and hyperparameter optimization supports the use of linear SVM. A. For each of the three univariate representations—A_{region}, $A_{feature}$, and A_{uni_combo}—the mean cross-validated balanced accuracy is shown per disorder using each of five different classifier types. Each dot corresponds to one model input type (e.g., left pericalcarine cortex in A_{region}) and lines connect model input types across classifiers per disorder to guide visual interpretation. The dashed horizontal lines indicate 50% balanced accuracy in all plots. **B.** For the same univariate representations as in **A**, the mean cross-validated balanced accuracy is shown for the linear SVM classifier without hyperparameter optimization (i.e., explicitly setting $C = 1$ and applying inverse probability weighting; green) or with hyperparameter optimization for the C parameter and sample weighting type in purple. The dashed horizontal lines indicate 50% balanced accuracy in all plots.

bioRxiv preprint doi: https://doi.org/10.1101/2024.01.10.573372; this version posted June 10, 2024. The copyright holder for this preprint (which was not certified by peer review) is the author/funder, who has granted bioRxiv a license to display the preprint in perpetuity. It is made available under aCC-BY 4.0 International license.

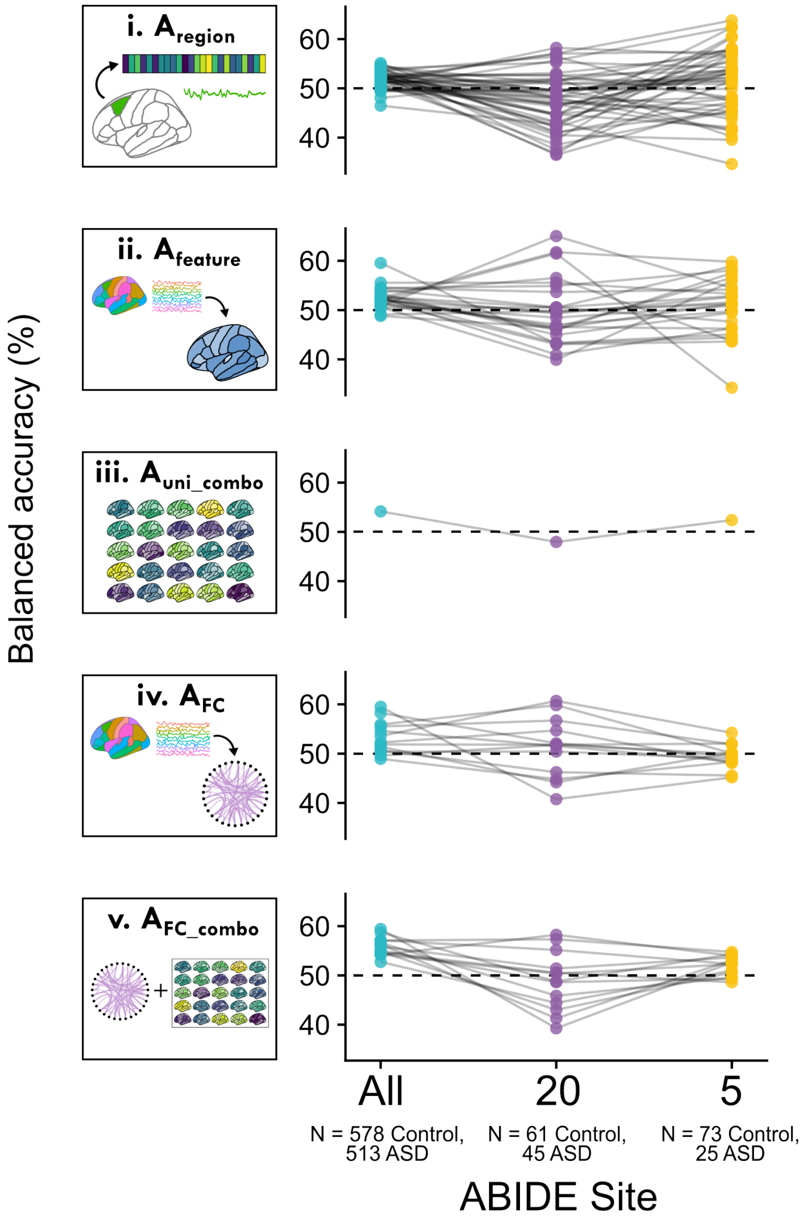

Figure S8. Classification performance is comparable within individual ABIDE consortium imaging sites. The mean cross-validated balanced accuracy is shown with the inclusion of participants from all ABIDE sites together (blue, $N = 1091$ participants) for each of **(i)** $\mathrm{A_{region}}$, **(ii)** $\mathrm{A_{feature}}$, **(iii)** $\mathrm{A_{uni\_combo}}$, **(iv)** $\mathrm{A_{FC}}$, and **(v)** $\mathrm{A_{FC\_combo}}$. The mean cross-validated balanced accuracy is also shown when we restricted classification analyses to each of the two largest ABIDE imaging sites: Site #20 (purple, $N = 106$ participants) and Site #5 (yellow, $N = 98$ participants). Each dot corresponds to one individual model (e.g., the Superior Frontal Gyrus in $\mathrm{A_{region}}$) and lines connect models across ABIDE site analyses to guide visual interpretation. The dashed horizontal line marks 50% balanced accuracy in all plots.

bioRxiv preprint doi: https://doi.org/10.1101/2024.01.10.573372; this version posted June 10, 2024. The copyright holder for this preprint (which was not certified by peer review) is the author/funder, who has granted bioRxiv a license to display the preprint in perpetuity. It is made available under aCC-BY 4.0 International license.

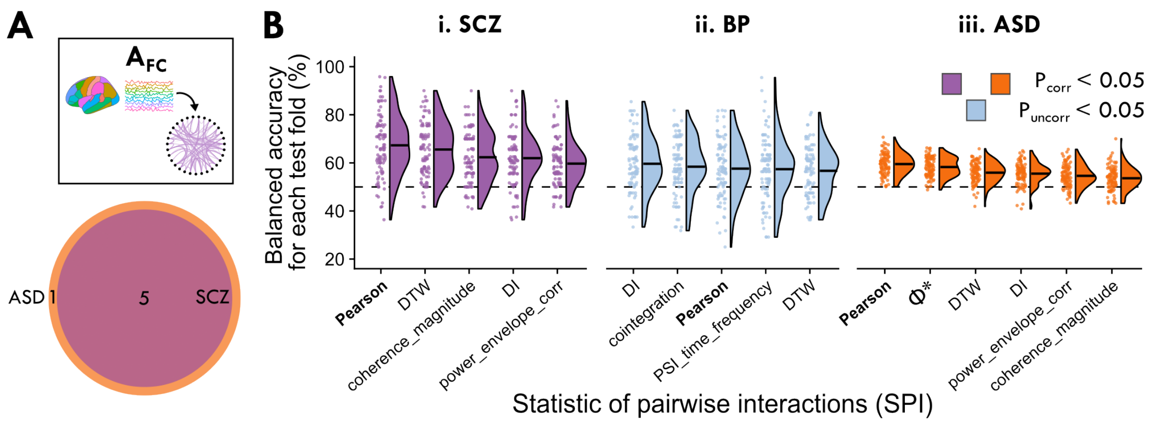

Figure S9. Representing brain activity as the set of all pairwise functional connectivity strengths, A_{FC}, can significantly distinguish cases from controls, with the classical Pearson correlation coefficient (capturing linear contemporaneous coupling) a top performing metric. **A.** We compared 14 statistics of pairwise interactions (SPIs) (from *pyspi* [29], cf. Sec. 4.2.2), as different ways of quantifying functional connectivity (FC) between pairs of brain regions. For a given SPI, each participant was represented by the set of corresponding FC values (calculated for each pair of brain regions), yielding a set of region–region pair values that can be stored as a one-dimensional vector per participant. These vectors were concatenated to yield a participant × region–pair matrix that formed the basis for case–control classification using a linear SVM. **B.** Classification results are shown as a heatmap, with rows representing SPIs that yielded significant balanced accuracy ($p_{\mathrm{corr}} < 0.05$, corrected across 14 SPIs) in at least one disorder, and columns representing each of the four disorders. Of the 14 SPIs we evaluated, eleven significantly distinguished cases from controls in at least one disorder, and are plotted here. The Pearson correlation coefficient is annotated in boldface for easier reference. **C.** The SPI similarity score, $|\rho_{\mathrm{SPI}}|$, is visualized between each pair of the eleven SPIs from B as a heatmap, revealing six clusters of SPIs with similar behavior on the dataset (based on their outputs across all region–pairs and all disorders). As in B, the Pearson correlation coefficient annotation is shown boldface. **D.** The disorder similarity score, ρ_{disorder}, is depicted to compare the balanced accuracy values among all 14 SPIs between each pair of neuropsychiatric disorders; a large positive ρ_{disorder} indicates a strong positive Spearman correlation in case–control classification performance across the 14 SPIs in the given pair of disorders.

bioRxiv preprint doi: https://doi.org/10.1101/2024.01.10.573372; this version posted June 10, 2024. The copyright holder for this preprint (which was not certified by peer review) is the author/funder, who has granted bioRxiv a license to display the preprint in perpetuity. It is made available under aCC-BY 4.0 International license.

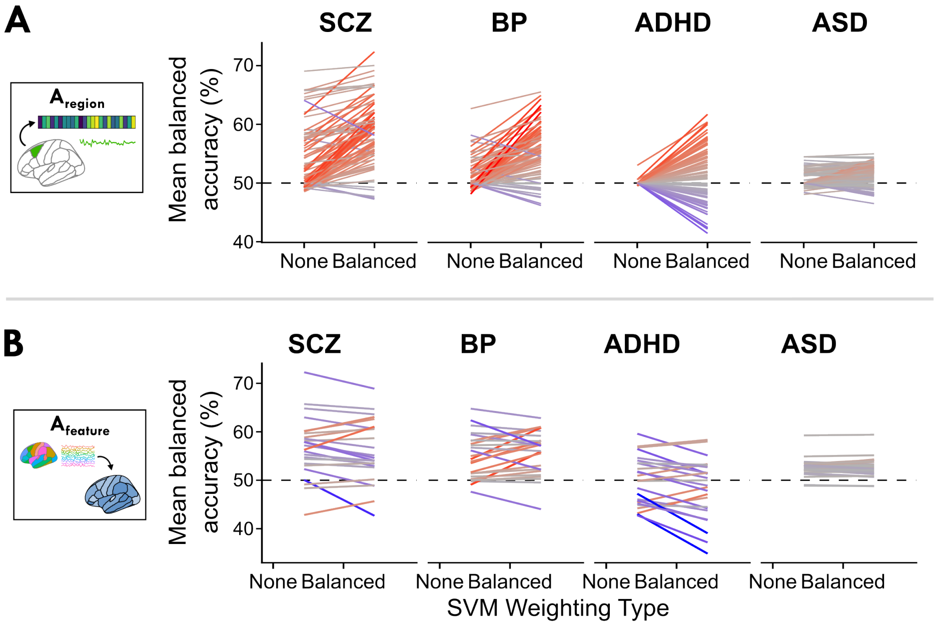

Figure S10. Comparing classification performance with versus without inverse probability weighting.
A. For each disorder, the mean balanced accuracy per brain region is shown with no weighting ('None') or inverse probability weighting ('Balanced'). Lines correspond to each of 82 brain regions for SCZ, BP, and ADHD and each of 48 brain regions for ASD. Colors are included as a visual aid to highlight the difference in performance between the two weighting types, with red corresponding to higher balanced accuracy with inverse probability weighting and blue corresponding to lower balanced accuracy with inverse probability weighting. **B.** For each disorder, the mean balanced accuracy per univariate time-series feature is shown with no weighting ('None') or inverse probability weighting ('Balanced'), as in A.

bioRxiv preprint doi: https://doi.org/10.1101/2024.01.10.573372; this version posted June 10, 2024. The copyright holder for this preprint (which was not certified by peer review) is the author/funder, who has granted bioRxiv a license to display the preprint in perpetuity. It is made available under aCC-BY 4.0 International license.

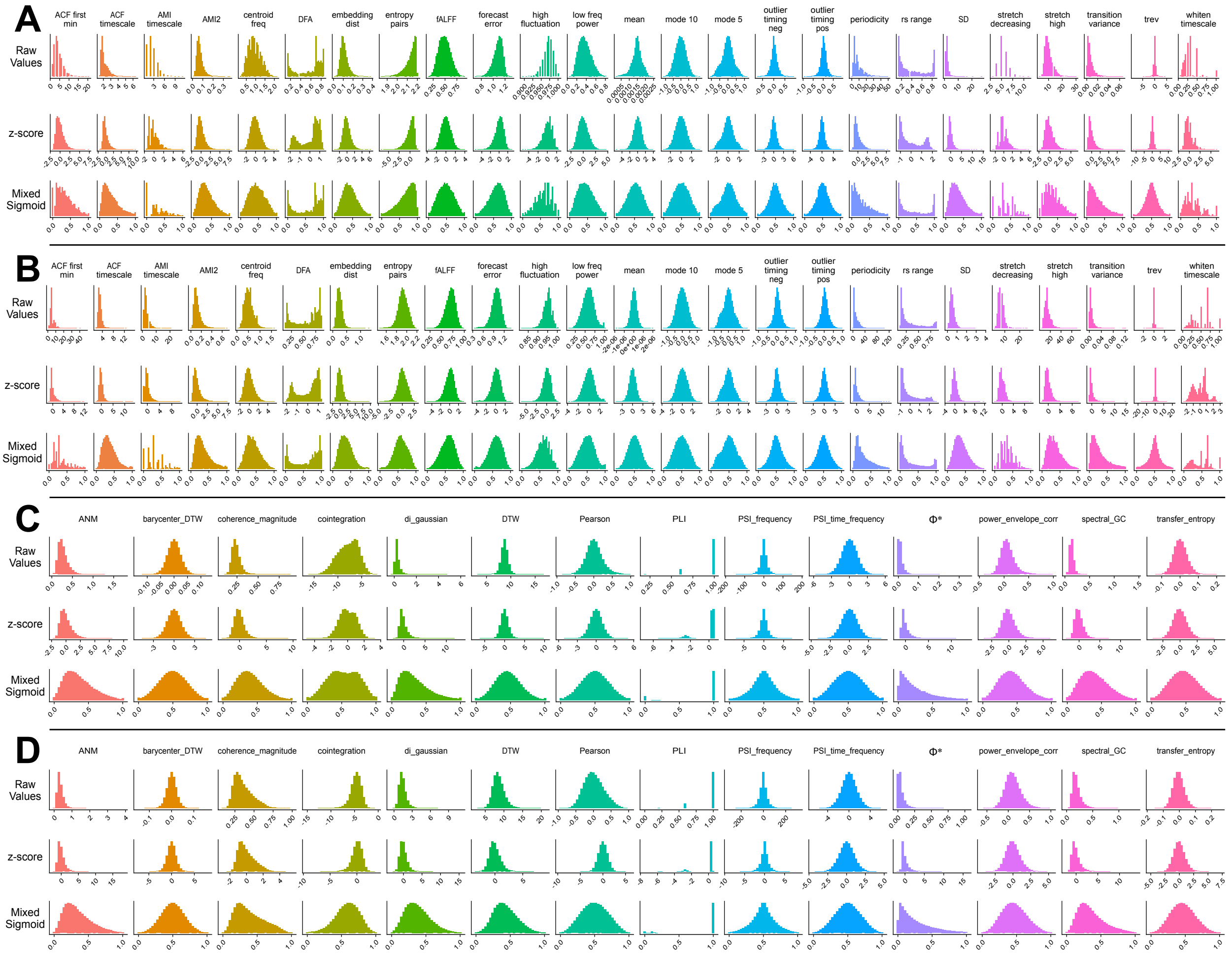

Figure S11. Comparing normalization methods supports the use of the outlier-robust mixed sigmoid method. Univariate time-series feature values were concatenated from all brain regions, with the resulting distributions depicted for all participants in the UCLA CNP cohort (**A**) or ABIDE cohort (**B**) with no normalization (upper row), z-score normalization (middle row), and outlier-robust mixed sigmoid normalization (bottom row; see Methods Sec. 4.3.1 for description). Pairwise SPI feature values were concatenated from all region–region pairs, with the resulting distributions depicted for all participants in the UCLA CNP cohort (**C**) or ABIDE cohort (**D**) with no normalization (upper row), z-score normalization (middle row), and outlier-robust mixed sigmoid normalization (bottom row).

bioRxiv preprint doi: https://doi.org/10.1101/2024.01.10.573372; this version posted June 10, 2024. The copyright holder for this preprint (which was not certified by peer review) is the author/funder, who has granted bioRxiv a license to display the preprint in perpetuity. It is made available under aCC-BY 4.0 International license.

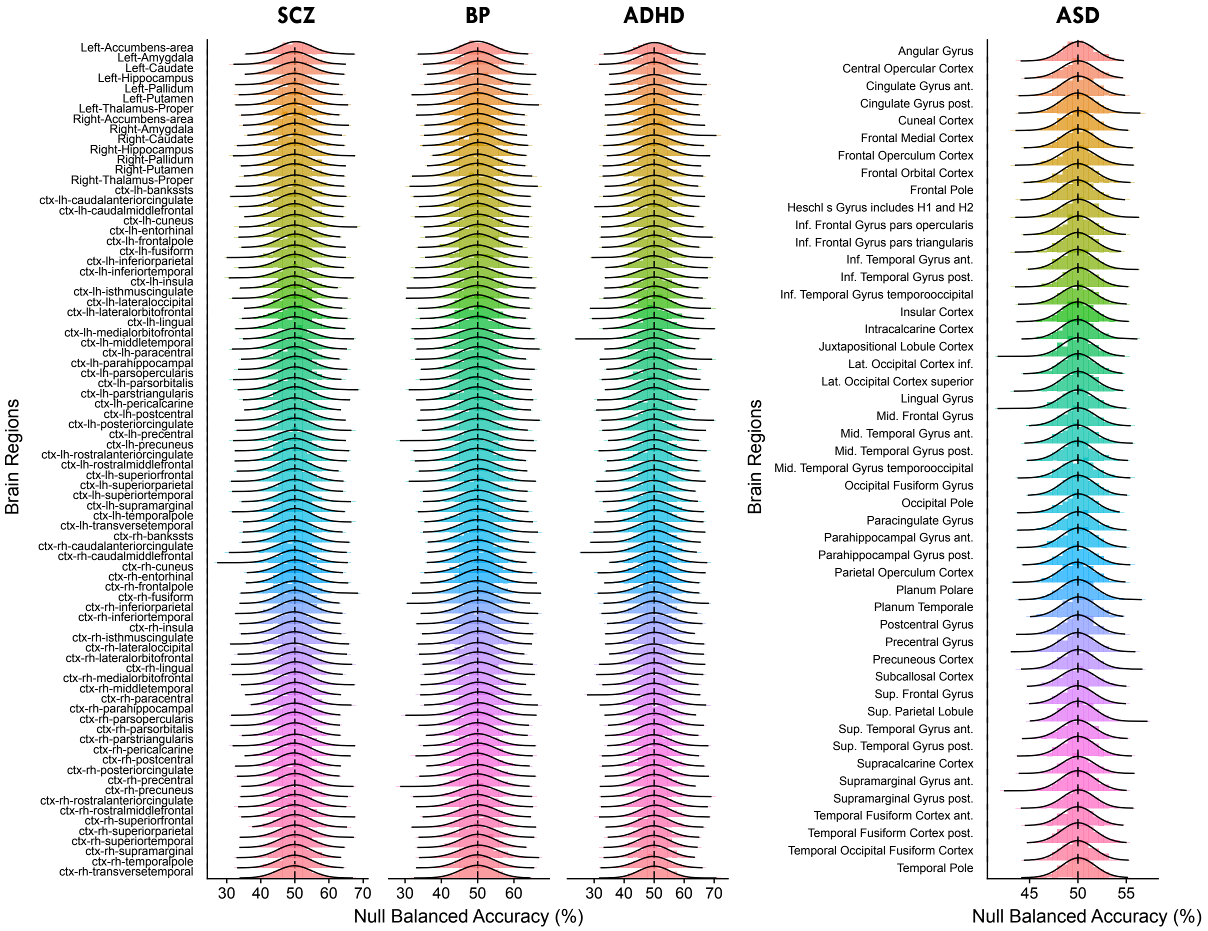

Figure S12. Null balanced accuracy metrics are approximately normally distributed across brain regions in all clinical cohorts. The distribution of 1000 null balanced accuracy values (cross-validated over 10 repeats of 10 folds each) is shown for each brain region by disorder. Empirical null values are visualized as histograms, with the probability density overlaid as black lines for the normal distribution based on the mean and standard deviation for each null distribution. These normal distributions were used to compute p-values for each brain region based on the corresponding probability density function; the same procedure was also applied for univariate and pairwise time-series features. ASD brain regions are plotted separately from the SCZ, BP, and ADHD brain regions as different parcellation atlases were analysed for UCLA CNP (Desikan-Killiany atlas) and ABIDE cohorts (Harvard-Oxford cortical altas). The dashed vertical line marks 50% balanced accuracy in all plots.
